# Naïve ants reach a food source quicker when encountering returning ants

**DOI:** 10.1038/s41598-025-02058-z

**Published:** 2025-05-16

**Authors:** Tomoko Sakiyama, Yuka Wada

**Affiliations:** Department of Information Systems Science, Faculty of Science and Engineering, Soka University, 1 Chome- 236 Tangimachi, Hachioji, Tokyo 192-8577 Japan

**Keywords:** *Lasius niger*, Foraging, Pheromone trails, Bidirectional flow, Neurophysiology, Population dynamics

## Abstract

Pheromone guidance of ant foraging activity is well described, but less is known about how the presence of inward ant traffic on the trail affects the behavior of naive nestmates and if these influences are dependent or independent of pheromone signaling. Here, we examined whether the flow of foraging *Lasius niger* ants coming from a feeder influences the movements of naive nestmates traversing a pheromone trail. We used a device that permitted individual ants to travel from the nest to a feeder via a single bridge under four conditions; unidirectional inward ant traffic, where naive foraging ants contacted only colony members returning from the feeder along a pheromone trail; foraging following a pheromone trail without ant traffic; foraging with neither a pheromone trail nor ant traffic; foraging with bidirectional ant traffic, where naive ants encountered other ants moving both toward the feeder and returning on the pheromone trail. The time required by inexperienced ants to reach the feeder was shorter in the unidirectional condition (returning from the feeder) than the other three conditions. These findings suggest that specific cues can regulate the movement of pheromone-guide foraging by ants.

**Supplementary Information:**

The online version contains supplementary material available at 10.1038/s41598-025-02058-z.

## Introduction

Colony-forming species like ants use pheromone signaling to maintain social organization and coordinate group activities such as foraging^1–4^. For instance, ants produce pheromone trails that act as transportation networks to food sources, thereby enhancing foraging efficiency^5–7^. Moreover, Argentine ants use trail networks adaptively under environmental changes^8^. They establish pheromone trails in response to changes in the location of high-quality food. Similarly, the degree of group symmetry-breaking among mass-recruiting Pharaoh ants appears to depend on food quality^9^.

Ants have been shown to modulate the behavioral response to pheromones or use pheromones adaptively for collective decisions according to environmental conditions and the activity of other conspecifics^10–14^. The ant *Lasius niger* downregulates pheromone deposition according to the behavior of nestmates. Czaczkes and colleagues (2013) reported that individuals decrease pheromone deposition rates when they encounter nestmates on a trail and at a food source, as presumably additional deposition is unnecessary^10^. Foraging ant groups also tend to select pheromone trails occupied by nestmates rather than empty trails, suggesting that the presence of nestmates acts as a social cue influencing the collective path choice of foragers^11^. Due to these environmental and social cues, ants do not always respond predictably to pheromone concentrations.

Some ant species are also reported to embed directional information in the geometry of pheromone trail networks^15^. Furthermore, encounters with nestmates can influence the directional behavior of individual foraging ants^16,17^. For example, a recent study of *L. niger* reported that naive ant foragers moved against ant traffic after entering the trail at a right angle^17^, suggesting that foragers place greater importance on a collective destination outside the nest (e.g., a food source). Supporting this notion, an agent-based model proposed by Sakamoto and Sakiyama (2022) indicated that ants moving against ant traffic would reach a goal location in a shorter time than those moving in the general direction of ant traffic^17^.

Considering these results, it is possible that the time taken by naive ants to reach a feeder is influenced by the flow of returning ants coming from a feeder. We hypothesized that naive ants will reach a food source (feeder) faster when contacting nestmates moving in the opposite direction than when encountering no other ants or bidirectional ant traffic. Although several studies have investigated the behavior of *L. niger* foragers in the presence of chemical cues^18,19^, these studies focused on performance differences between experienced and naive ants or performance differences depending on food quality^18,19^. In this study, we investigated the time required for individual naive *L. niger* ants to reach a food source as a measure of foraging efficiency without the aid of memory and under conditions where the presence of a pheromone trial and both the presence and direction of conspecifics could be controlled, thus creating four distinct conditions: no pheromone trail and no conspecifics, pheromone trail but no conspecifics, pheromone trail with conspecifics moving in the oppositive direction, and pheromone trail with conspecifics moving in both directions.

## Methods

### Experimental animals

Ten colonies of garden *L. niger* ants consisting of 400–700 workers and some brood items but no queen were collected from Soka University (See supporting dataset). This species was chosen for consistency and comparability with our previous study^17^. Colonies were housed in a plastic case (35 × 25 × 6 cm) filled with soil and containing a plastic petri dish with water-soaked filter paper and another with sucrose solution. These food and water sources were exchanged with fresh supplies every few days. The colonies were kept in the same laboratory where all experiments were conducted under controlled temperature (23 °C ± 2 °C) and humidity (70% ± 10%). Each colony was starved for 4–6 days prior to experiments to motivate foraging behavior.

### Experimental apparatus

**Fig. 1:**
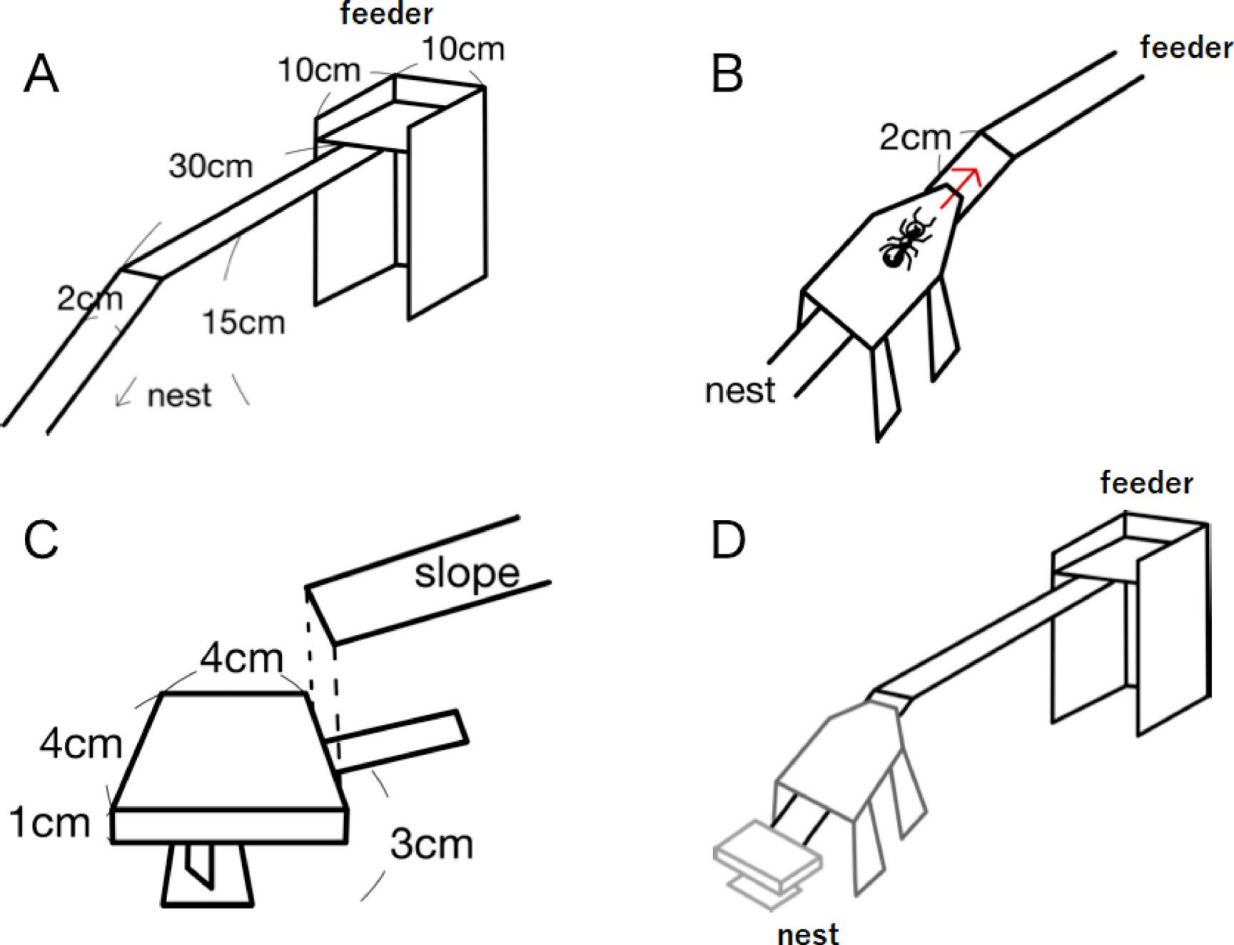
The experimental apparatus configurations used in different experiments. (**A**) A conduit between an ant nest and a feeder was established using an elevated bridge with entrance ramp (slope) extending down to the nest. Foraging thus required movement in the following direction: slope → bridge → feeding area → bridge → slope. (**B**) The removable connecting device used to introduce target ants onto the slope. Target ants were introduced in the middle of the slope with a 2 cm gap from the start of the elevated bridge section. The slope was covered by this device to minimize interference with the ant trail (if present). (**C**) Rat-guard structure used only in the main experiment to restrict ant movement from the nest onto the bridge but still allow return from the feeder (i.e., unidirectional inward ant traffic). The rat-guard structure was installed just prior to introducing the first target ants with the connecting device. (**D**) Complete experimental apparatus for the main experiment.

The experimental system used to create the four experimental conditions described below is shown in Fig. 1 and Supplementary Fig. 1. The nest site was connected to a feeder containing sucrose via a bridge (Fig. 1A) and connecting device (Fig. 1B). The assembly shown in Figs. 1A, B was used in the three control experiments in which target (test) ants encountered a pheromone trail but no other nestmates (control experiment 1), no pheromone trail and no other nestmates (control experiment 2), or a pheromone trail and nestmates traveling both from the nest to the feeder and from the feeder to the nest (control experiment 3). For the main experimental condition during which target ants encountered only nestmates traveling in the opposite direction (from feeder to nest), a rat-guard structure (Figs. 1C, D) was placed between the nest and bridge to prevent ants from leaving the nest.

In detail, the bridge included an elevated region (length: 30 cm, width: 2 cm, height: 15 cm) connected directly to the feeder and a sloped region (length: 25.5 cm, width: 2 cm) projecting down to the nest. The connecting device (length: 14 cm, width: 3.5 cm, height: 10 cm) was set on the slope 2 cm away from the entrance to the elevated section (Fig. 1B). Individual ants were introduced to the slope using this connecting device and allowed to spontaneously climb the slope to reach the elevated bridge section and feeder. The point at which this device contacted the slope was narrow, so it did not allow easy entry by ants returning to the nest from the feeder (if present) (See Fig. 1B, D). The rat-guard structure use in the main experiment to block outward movement of nontarget ants was attached immediately before the first target ants were introduced using the connecting device. Because of the rat-guard structure, ants climbing up from the nest make U-turns, whereas fed ants try to climb down from the slope and return to the nest. During all four experiments, the main apparatus was covered on all sides by white plastic walls so that nontarget ants coming from the nest had to reach the slope region without the use of visual information.

Each colony was used for experiments “main followed by control 1” or “control 2” or “control 3” within one day. Ants exploring the case outside the nest were collected prior to each experiment as test subjects as with a previous study so as not to interact with returning ants from the feeder within their nest^17^. Thus, all target ants were naive to the test apparatus and the presence of a feeder. These ants were then excluded from the colony and further experiments after a single trial.

### Main experiment—pheromone trail with unidirectional inward ant traffic toward the feeder

**Fig. 2:**
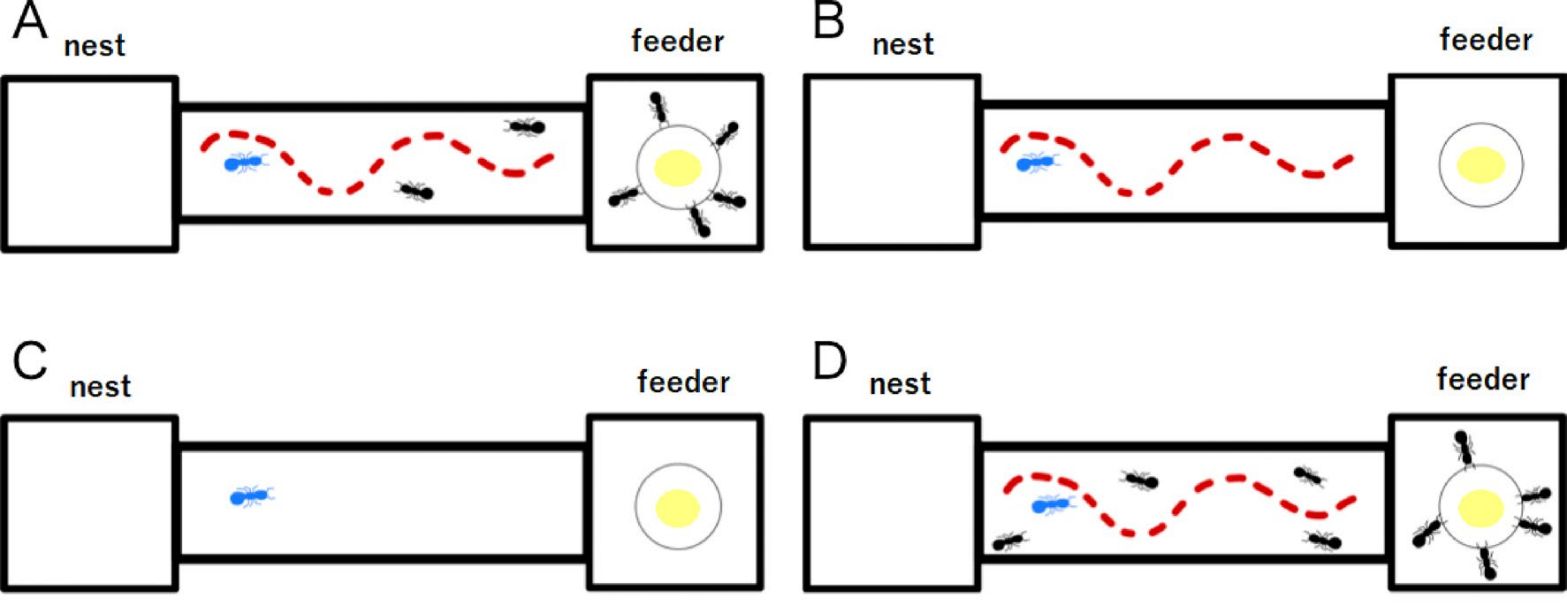
Illustrations of each experimental condition. (**A**) Main experiment (pheromone trail with unidirectional inward ant traffic). Target ants (blue) only encounter foraging ants (black) returning from the feeder. The pheromone trail is indicated by the red dotted line. (**B**) Control experiment 1 (pheromone trail with no ant traffic). (**C**) Control experiment 2 (neither pheromone trail nor ant traffic). (**D**) Control experiment 3 (pheromone trail with bidirectional ant traffic).

In the main experiment, about 10 to 20 target ants were collected randomly from the colony and isolated. Then, a droplet of sucrose solution was introduced on the feeder, and nontarget ants in the nest were allowed to freely form an ant trail and forage for 10 min (Fig. 2A). During this time, both outward traffic (from nest to feeder) and inward traffic (from feeder to nest) were observed. Thereafter, to prevent further movement of nontarget ants from the nest to the feeder, we connected the rat-guard structure to the end of the slope and used a brush to remove ants already near the end of the slope. We then introduced individual target ants to the slope one at a time via the connecting device and recorded movements by video capture (described in the ‘Data analyses’ section). After the target ant reached the food source, the next target ant was introduced following the same procedure. This process was repeated thereafter.

### Control experiment 1—pheromone trail with no ant traffic

In the first control experiment, about 10 to 20 target ants were collected randomly from the colony and isolated. Individual target ants were exposed only to the preestablished pheromone trail without any current ant traffic (Fig. 2B). This experiment was conducted after the end of the main experiment to ensure robust pheromone trail formation. This is because the returning ants were reorienting towards their nests from the feeder until just before the end of the main experiment. A single target ant was introduced to the slope via the connecting device and movements were recorded. In addition, a droplet of sucrose solution was placed on the feeding area as in the main experiment to control for the influence of food odor. After the target ant reached the food source, the next target ant was introduced following the same procedure. This process was repeated thereafter.

### Control experiment 2—neither pheromone trail nor ant traffic

In control experiment 2, about 10 to 20 target ants were collected randomly from the colony and isolated. Individual target ants were allowed to move on the main bridge in the absence of other nestmates or a pheromone trail (Fig. 2C). We then introduced a single target ant to the slope via the connecting device. After each ant, any pheromones deposited by the target ant were cleaned with ethanol. As in control experiment 1, a droplet of sucrose solution was placed at the feeding area to control for the influence of food odor. After the target ant reached the food source, the next target ant was introduced following the same procedure. This process was repeated thereafter.

### Control experiment 3—pheromone trail with bidirectional ant traffic

In control experiment 3, about 10 to 20 target ants were collected randomly from the colony and isolated. Individual target ants were allowed to interact with both outward- and inward-traveling ants on a pheromone trail (Fig. 2D). To this end, individual target ants were paint-marked to distinguish them from other ants on video recordings. Then, a droplet of sucrose solution was placed on the feeder and bidirectional ant traffic was created by connecting the nest to the feeder. As in the main experiment, approximately 10 min were required to form a line of ants. However, unlike the main experiments, we did not connect the rat-guard structure to the end of the slope before introducing a single target ant via the connecting device. After the target ant reached the food source, the next target ant was introduced following the same procedure. This process was repeated thereafter.

### Data analyses

All four experiments were video recorded from above the bridge at 29.97 fps using a high-definition digital camera (Panasonic, HC-V480MS). First, we measured the time required for individual target ants to reach the feeder (defined as the time from exiting the connecting device to the head crossing the distal edge of the bridge). In all four experiments, trials were eliminated if the target ant retreated to the nest or otherwise never entered the main section of the bridge. We also eliminated trials if the target ant fell from the bridge or made a U-turn and returned to the nest after entering the main bridge section. Those eliminated ants might not be in a motivational state to go foraging.

In the main experiment (unidirectional inward ant traffic) and control experiment 3 (bidirectional traffic), a target ant was considered interacting with other ants if the two ants were within 2 cm of each other (the bridge width). This threshold was adopted because it was sometimes difficult to determine whether ants made direct contact, especially when traveling in the same direction. It is worth noting that the results remained qualitatively unchanged when a 1 cm threshold was applied. Trials in which there were no interactions with other ants before reaching the feeder were excluded. In the main experiment, returning ants sometimes made U-turns and temporarily faced toward the feeder. If these ants, facing toward the feeder, entered within a 2 cm radius of the target ants, we excluded these trials, as the target ants in the main experiment were only allowed to interact with returning ants heading toward the nest. In control experiment 3, trials in which only outgoing ants entered within a 2 cm radius of a target ant before reaching the feeder were excluded (i.e., trials with no interactions with returning ants). Similarly, trials where only returning ants entered within a 2 cm radius from a target ant before reaching the feeder were excluded. Thus, in all control experiment 3 trials included in the analyses, target ants interacted with both leaving and returning ants at least once. An ant was considered returning to the nest if the head position was on the nest side of the bridge slope relative to gaster position. Otherwise, that ant was considered outgoing (Figure S2). This classification was conducted only when an interaction was observed.

We also performed a detailed motion analysis of target ants on the bridge. Individual images were created every 0.1 s (10 frames/s) using the recorded video, and coordinates were acquired using ImageJ (Ver.1.53t^20^). Coordinates of the target individual were measured according to head position and the following parameter calculated using the C language. We obtained the angular change rate every 0.1 s (the change of direction between two trajectory steps). Using the position of the ant’s head, individual angle information was measured relative to the direction toward the feeder (with positive direction of the x-axis considered as 0° and counterclockwise was defined as the positive direction (Figure S2)). Based on the angle information between two consecutive head position of a target ant, the absolute angular change rate was measured. We then averaged those values for each individual to obtain the mean angular displacement for individuals. The mean value of the time series data for each individual ant was considered as the analysis data for that ant. In addition, we calculated the travel distance every 0.1 s. We averaged those values for each individual to obtain the mean travel distance for individuals.

### Statistics

All statistical analyses were conducted using R (ver. 4. 2. 1^21^). Please see Table S1 for GLMM information.

### Time measurement/motion analysis on the bridge

A generalized linear mixed model (GLMM) considering the colony effect as random was used to compare time measurements and motion analysis on the bridge (angular displacement and speed (travel distance per 0.1 s)) between the main experiment and control experiments. A multiple comparison test was then conducted, which compares mean values from several control groups against the mean values of a treatment group (the main experiment in this case) to determine whether there was a difference (Benjamini-Hochberg correction).

### Effects of ant interactions on time measures

The number of interactions between targets and nestmates may influence travel times, thereby accounting for differences between experimental conditions. To examine this possibility, we used the GLMM considering the colony effect as random to estimate the correlation between travel time and number of interactions in the main experiment and control experiment 3, and to compare travel time between the main experiment and control experiment 3.

### Ethics approval

No licenses or approvals were required to conduct this work.

## Results

### Effects of ant traffic direction on time to reach the feeder

**Fig. 3:**
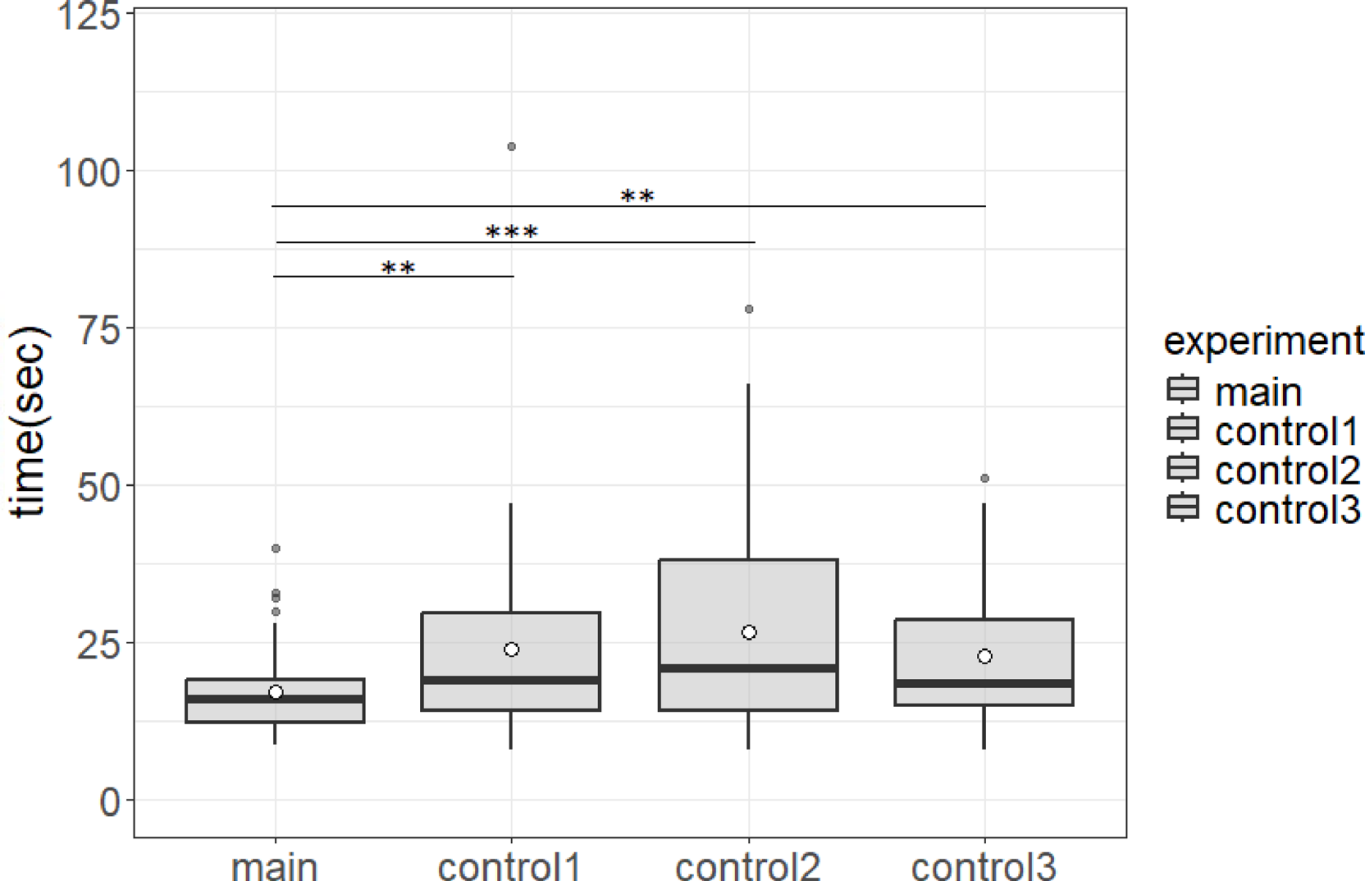
Mean travel times for the four experimental conditions. The ○ symbol indicates the mean value and the thick horizontal line in the box indicates the median value. The upper and low limits of the box are the 75th and 25th percentiles, respectively, while the upper and lower ends of the whisker indicate the maximum and minimum values, respectively. The small filled dots are outliers. The travel times of target ants to the feeder were significantly reduced in the main experimental condition (with nestmate flow in the opposite direction). ***p < 0.001 and **p < 0.01. The four experimental conditions are Main (pheromone trail with unidirectional inward ant traffic), Control 1 (pheromone trail with no ant traffic), Control 2 (neither pheromone trail nor ant traffic) and Control 3 (pheromone trail with bidirectional ant traffic). Box plot showing 42 data points in the main experiment and 50, 43, and 36 in control experiments 1, 2, and 3, respectively.

We compared the time required for individual target ants to reach the feeder under the four experimental conditions (Fig. 3). A total of 42 target ants from four colonies were used in the main experiment, 50 ants from four colonies in control experiment 1, 43 ants from four colonies in control experiment 2, and 36 ants from ten colonies in control experiment 3. The average travel time of target ants to the feeder was significantly shorter in the main experimental condition with movement of nestmates only in the opposite direction (toward the nest) compared to control experiment 1 with a preestablished pheromone trail but no nestmate encounters (17.13 ± 7.12 s vs. 23.88 ± 15.87 s mean ± SD, *p* < 0.01). Similarly, average travel time to the feeder was shorter in the main experiment than control experiment 2 with no nestmate encounters or pheromone trail (17.13 ± 7.12 s vs. 26.74 ± 16.66 s, *p* < 0.001), and was shorter than control experiment 3 in which target ants encountered nestmates moving in both directions (17.13 ± 7.12 s vs. 22.87 ± 11.26 s, *p* < 0.01).

### Effects of experimental condition on target ant speed and directionality (meandering)

**Fig. 4:**
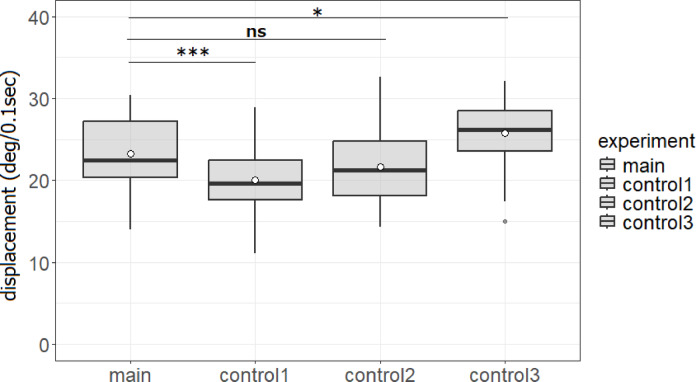
Average angular displacement (deg/ 0.1 s) of the target ant under the four experimental conditions. The symbols and box-plot structure are as described in Fig. 3. Angular displacement was significantly greater in control experiment 3 with bidirectional ant flow. *p < 0.05 and ***p < 0.001, ns: nonsignificant. The four experimental conditions are Main (pheromone trail with unidirectional inward ant traffic), Control 1 (pheromone trail with no ant traffic), Control 2 (neither pheromone trail nor ant traffic) and Control 3 (pheromone trail with bidirectional ant traffic). Box plot showing 42 data points in the main experiment and 50, 43, and 36 in control experiments 1, 2, and 3, respectively.

**Fig. 5:**
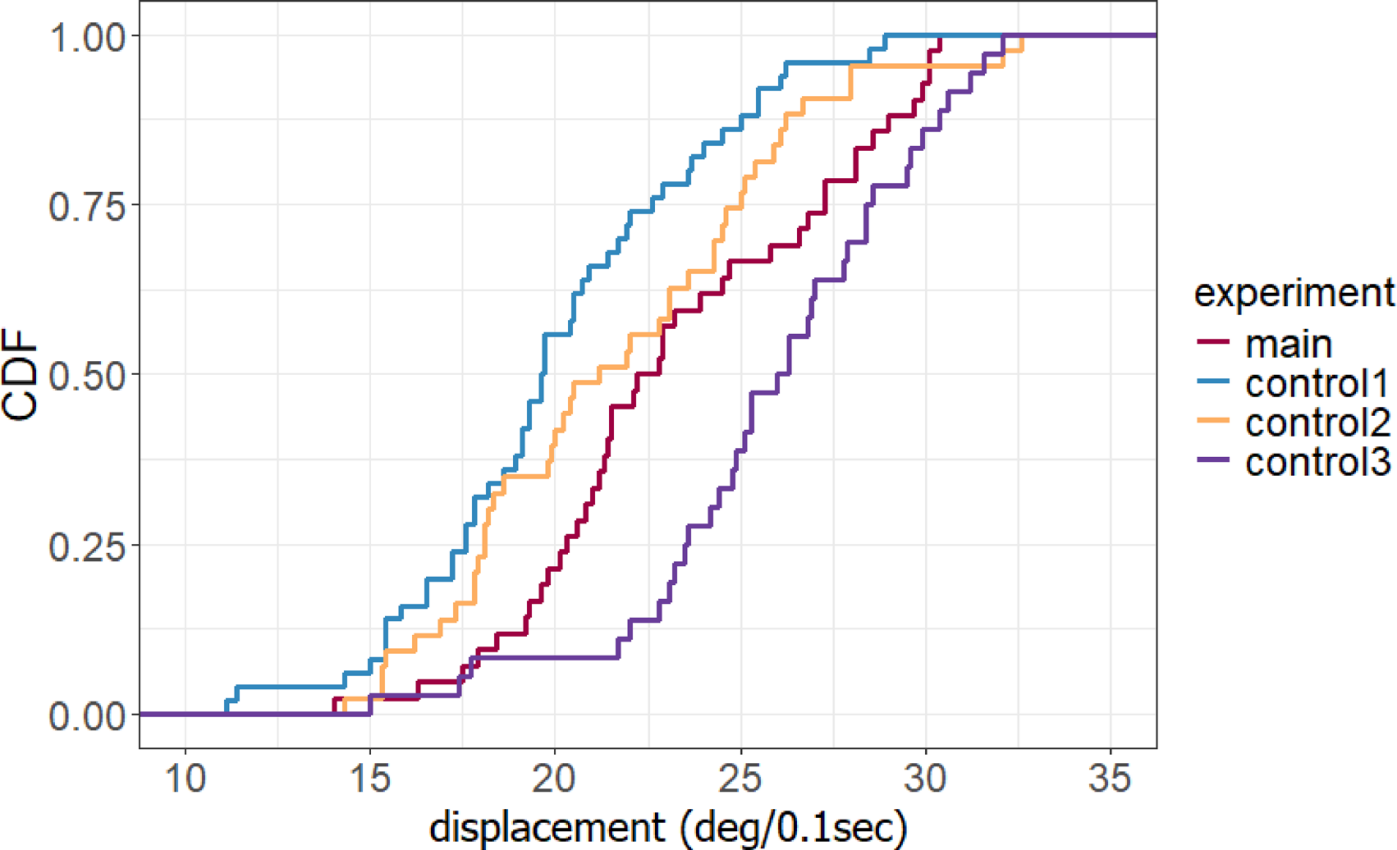
Cumulative distribution functions (CFD) for angular displacement (deg / 0.1 s) per 0.1 s in each experimental condition.

There was a significant difference in the average angular displacement between the main experiment with unidirectional inward traffic of nestmates and control experiment 1 with pheromone signaling but no nestmate encounters (23.30° ± 4.25°/0.1 s vs. 20.00° ± 3.96°/0.1 s, *p* < 0.001) (Fig. 4). On the contrary, there were no significant differences in the average angular displacement between the main experiment and control experiment 2 with neither pheromone signaling nor nestmate encounters (23.30° ± 4.25°/0.1 s vs. 21.69° ± 4.45°/0.1 s, *p* = 0.081), possibly because ants are more likely to meander in the absence of pheromone sensing compared to when pheromones are present^19^. However, compared to the main experiment, the average angular displacement was greater in control experiment 3 with bidirectional nestmate encounters (23.30° ± 4.25°/0.1 s vs. 25.81° ± 3.96°/0.1 s, *p* < 0.05 (*p* = 0.026), likely because nestmates approaching from multiple directions encouraged greater meandering. Cumulative distribution functions regarding angular displacements are shown in Fig. 5.

**Fig. 6:**
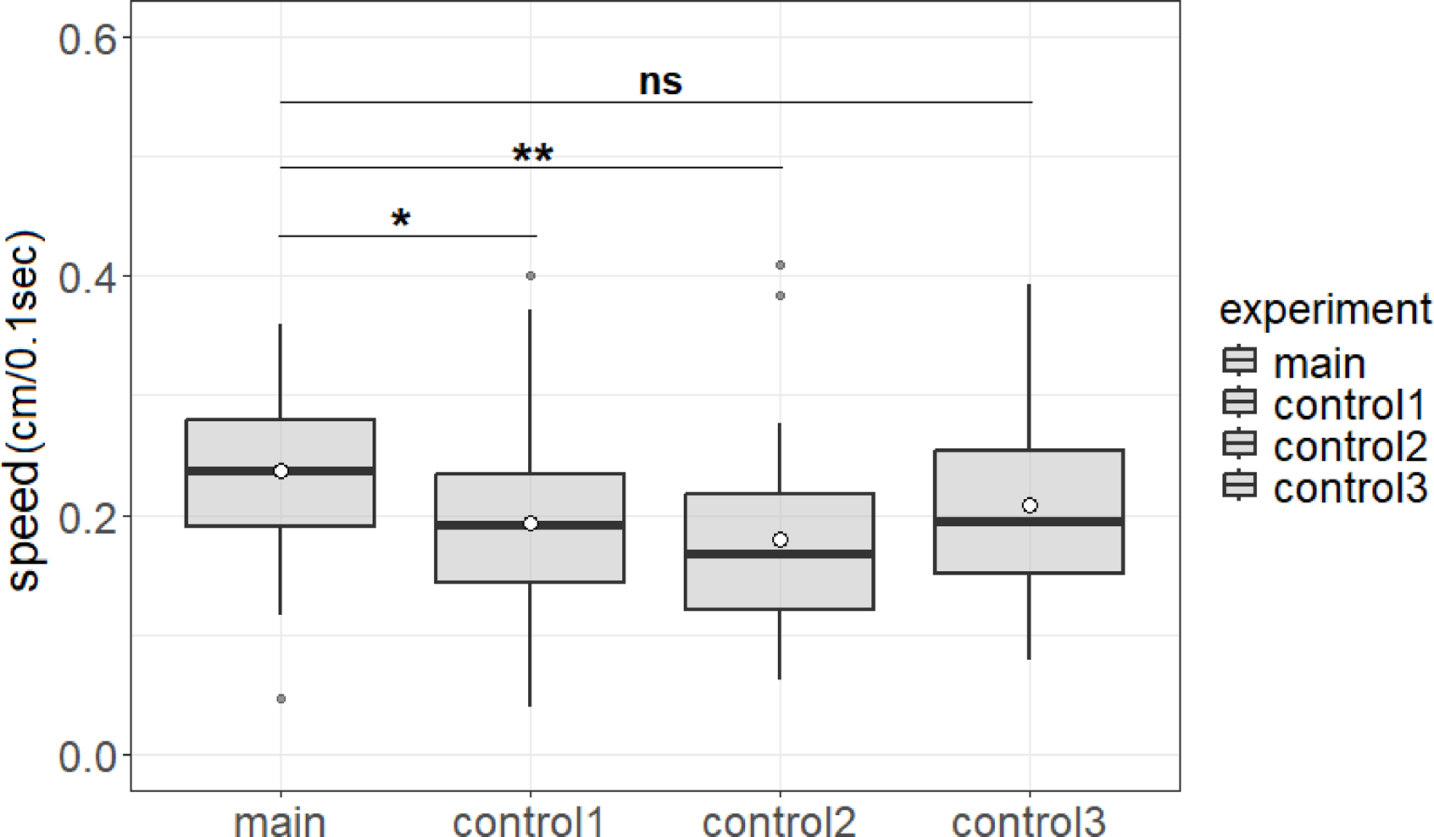
Average speed (distance traveled by target ants per 0.1 s) in the four experimental conditions. The symbols and box-plot structure are as described in Fig. 3. Speed was significantly greater in the main experimental condition with unidirectional inward ant traffic. *p < 0.05 and **p < 0.01, ns: nonsignificant. The four experimental conditions are Main (pheromone trail with unidirectional inward ant traffic), Control 1 (pheromone trail with no ant traffic), Control 2 (neither pheromone trail nor ant traffic) and Control 3 (pheromone trail with bidirectional ant traffic). Box plot showing 42 data points in the main experiment and 50, 43, and 36 in control experiments 1, 2, and 3, respectively.

**Fig. 7:**
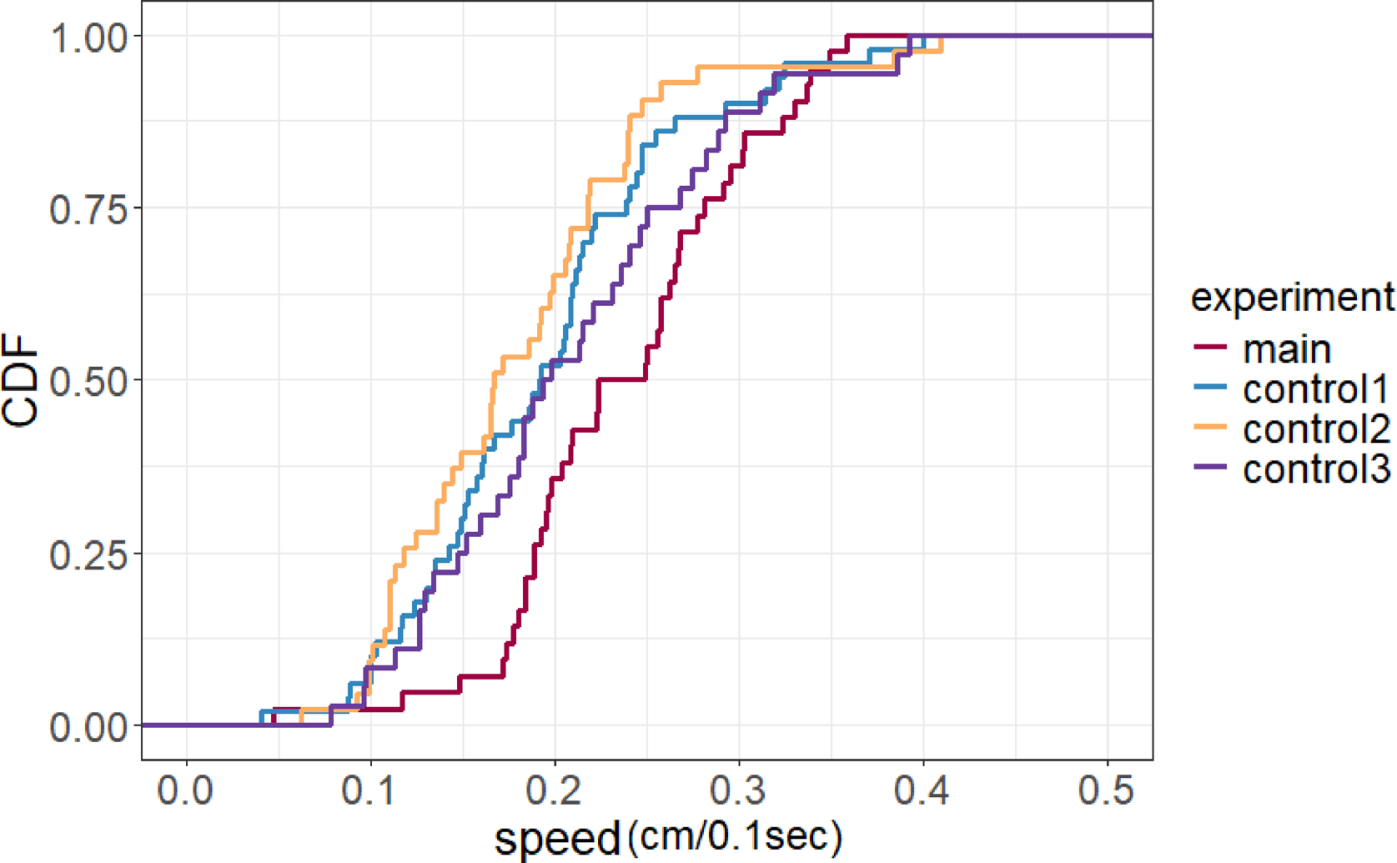
Cumulative distribution functions (CDF) for distance traveled per 0.1 s in each experimental condition.

The speed (the distance traveled per 0.1 s by target ants) was also significantly greater during the main experiment compared to control experiment 1 (0.24 ± 0.067 cm/0.1 s vs. 0.19 ± 0.075 cm/0.1 s, *p* < 0.05 (*p* = 0.013)) and control experiment 2 (0.24 ± 0.067 cm/0.1 s vs. 0.18 ± 0.072 cm/0.1 s, *p* < 0.01). However, no significant difference was found regarding the speed between the main experiment and control experiment 3 (0.24 ± 0.067 cm/0.1 s vs. 0.21 ± 0.078 cm/0.1 s, *p* = 0.095) (Fig. 6). Cumulative distribution functions are shown in Fig. 7. As shown in the results of time measurement, the time taken for ants in the main experiment to reach the food source was significantly shorter compared to those in control experiment 3. In contrast, no significant difference was observed in speed between the two groups. However, given the observed differences in angular displacement between the groups, it is likely that these differences contributed to the variation in the time required to reach the food source.

### Effects of interacting with ants on the trail

Finally, we investigated the effects of nestmate interactions on travel time in the main experiment and control experiment 3. There was no significant correlation between travel time and number of interactions in either the main experiment or control experiment 3 (main experiment: *p* = 0.073, control experiment 3: *p* = 0.23). There was also no significant difference in the number of interactions between the main experiment and control experiment 3 (4.95 ± 4.55 per trial vs. 5.92 ± 3.66 per trial, *p* = 0.33, ns). Therefore, a greater number of nestmate interactions cannot account for the longer travel time under bidirectional ant flow compared to unidirectional inward ant flow.

In fact, no strong correlations were found between the mean travel times and angular displacement, nor between the number of interactions and angular displacement in either of the experiments (Figure S3 and S4). In comparison to the other variables, only the number of interactions and angular displacement in the control experiment 3 exhibit a weak positive correlation (Figure S4B). This could be attributed to the fact that increased wandering behavior in ants is more likely to result in encounters with other individuals or that the collision with other ants contributes to their increased meandering behavior.

## Discussion

We analyzed the foraging movements of individual *L. niger* ants under several distinct experimental conditions and found that the time required to reach a food source was reduced when encountering exclusively ants coming from the feeder along a pheromone trail compared to no encounters. Furthermore, individual target ants in the bidirectional movement condition took longer to reach the food source than individual target ants in the unidirectional inward traffic condition (returning from the food source). The target ants in the main experiment took less time to reach the food source than the ants in the other conditions, perhaps due to encounters with nestmates returning from the feeder, possibly driven by motivation from detecting food residues on the mandibles of returning ants or from motivational signaling by those ants. Thus, this tendency may be more pronounced when the target ants encounter only conspecifics returning from the feeder (under the unidirectional inward traffic condition)^20^.

In addition, as evidenced by the results of the angular displacement analysis, target ants only encountering nestmates coming from the feeder made fewer or smaller angular deviations than target ants encountering nestmates moving in both directions. Interestingly, however, in both the main experiment and control experiment 3, the frequency of contacts, whether high or low, did not appear to have a significant effect on the time taken to reach the feeding site, which is consistent with the previous study^23^.

Moreover, while target ants meandered more under the unidirectional inward traffic condition than the lone condition (control condition 1), travel time was still shorter, suggesting that target ants moving in the opposite direction to nestmates maintained an efficient speed. Also in accord with our findings, outward-traveling naive ants contacting inbound scout ants exhibited higher angular velocities than ants reaching an experimental arena devoid of other ants^18^.

Poissonnier and colleagues (2019) reported that, at low densities, a clear linear relationship exists between Argentinian ant density and flow, whereas at high densities, the flow becomes constant, and no congestion is observed, indicating that foraging behavior is not a passive physical process dependent on density^24–27^ and cannot be explained by simple particle system dynamics^28–31^. In fact, ants do not behave like a solid^28–30^. Particle-to-particle collisions naturally occur during ant foraging. Studies using a different ant species have reported thathead-on collisions lead to a decrease in speed, potentially influencing foraging efficiency and suggesting that excessive overcrowding may eventually obstruct movement^32^. In such cases, however, *L. niger* foragers may still use pheromone signaling and other cues for traffic regulation to maintain a high rate of food return^33,34^. For instance, according to Dussutour and colleagues (2004), head-on collisions between ants at a branch junction may maintain a high rate of food return to the nest: at high levels of crowding, an additional trail is established before traffic volume is impacted.

These adaptations could be influenced by alterations in the pheromone response. A recent study found that ants changed their strategy from relying on memory to following pheromone trails when in the presence of nestmates that had access to better food sources than those encountered by experienced nestmates^12^. Thus, it is essential to combine pheromone guidance with other cues, like the behavior of nestmates.

This study exclusively focuses on naive ants heading toward the feeder and examines how encounters with ants returning to the nest influence their movement speed. There may be a greater number of ants on their way back to the nest than heading toward the feeder, potentially due to the presence of multiple foraging routes that converge or as a result of the initial phase of pheromone recruitment. In particular, in the latter scenario, the ability of naive ants to reach the feeder rapidly during the initial phase of pheromone recruitment may enhance the subsequent foraging efficiency of the entire colony.

Further investigations are warranted to examine the effects of nestmate speed and feeding status as well as the relative contributions of pheromones, memory, internal state, and other environmental cues on foraging efficiency^35–37^.

## Electronic supplementary material

Below is the link to the electronic supplementary material.

Supplementary Material 1.

Supplementary Material 2.

## Data Availability

All data are available in the supporting file.
