# Supplementary material for: Naïve ants reach a food source quicker when encountering returning ants
Source: Sci Rep. 2025 May 16;15:17008. doi: 10.1038/s41598-025-02058-z (PMC12084366; doi:10.1038/s41598-025-02058-z)
Supplement: Supplementary file 1 — Supplementary Material 1. [file 41598_2025_2058_MOESM1_ESM.docx]

Results – including non-reaching ants:

As reported in the main manuscript, in some trials, the target ant fell from the bridge or made a U-turn and returned to the nest after entering the main bridge section. Here, we included those ants to the analysis of travel time reaching for the feeder. We recorded the time from release until falling or turning around and recorded the displacement from the entry point to the site of falling or turning.

Here, we calculated the normalized time for those non-reaching ants as follows:

Normalized time (s) = (recorded time (s) / recorded displacement (cm)) × 30 cm

The average travel time of target ants to the feeder was significantly shorter in the main experimental condition compared to the other three control experiments (vs. control 1: 17.22 ± 7.10 s vs. 23.88 ± 15.87 s, *p* < 0.01, vs. control 2: 17.22 ± 7.10 s vs. 26.74 ± 16.66 s, *p* < 0.001, 17.22 ± 7.10 s vs. 22.89 ± 11.61 s, *p* < 0.01).


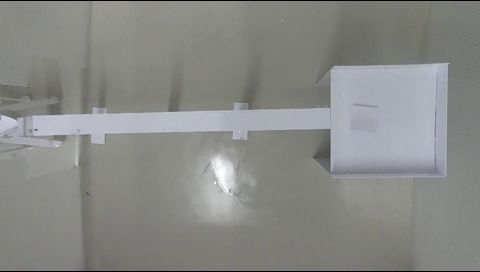


**Figure S1. A photograph of the main apparatus (acquired at the start of a control experiment 2 trial).**


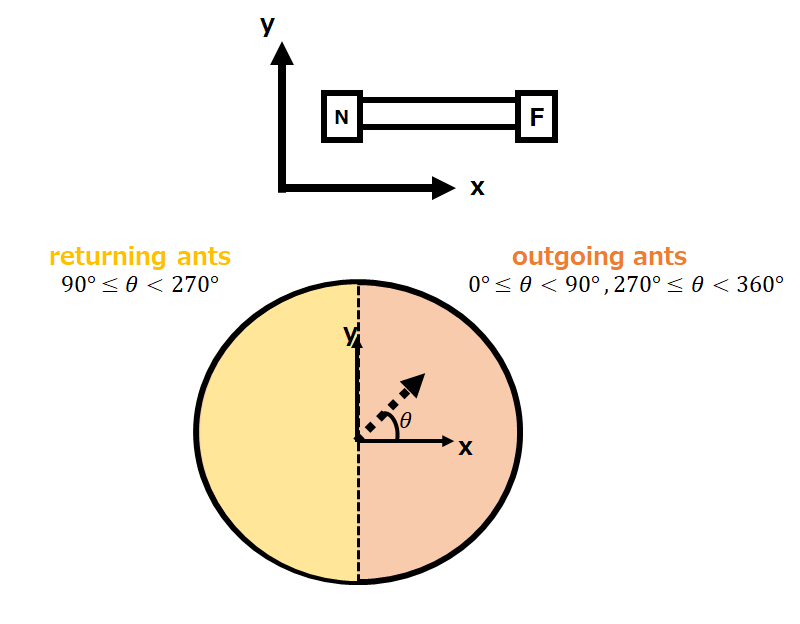


**Figure S2. Illustration of the definition used to distinguish ants traveling away from the nest and toward the feeder (outgoing ants) and ants returning to the nest (returning ants).** The dashed arrow indicates the head direction of a target ant, while N and F indicate the nest and feeder, respectively. If $\theta$ is between $0^{^{\circ}}\leq\theta\leq{90}^{^{\circ}}$ or ${270}^{^{\circ}}\leq\theta<{360}^{^{\circ}}$, the target ant is outgoing, while if $\theta$ is between ${90}^{^{\circ}}\leq\theta<{270}^{^{\circ}}$, the target ant is returning to the nest.

| **Analysis** | **The data distribution** | **Model equation** |
| --- | --- | --- |
| Effects of ant traffic direction on travel time for reaching the feeder | Gamma | ${log(\mathbb{E}[time}_{ij}])=\beta_{0}+\beta_{1}{experiment}_{i}+u_{j}$ |
| Effects of experimental condition on target ant directionality | Gamma | ${log(\mathbb{E}[angle}_{ij}])=\beta_{0}+\beta_{1}{experiment}_{i}+u_{j}$ |
| Effects of experimental condition on target ant speed | Gamma | ${log(\mathbb{E}[speed}_{ij}])=\beta_{0}+\beta_{1}{experiment}_{i}+u_{j}$ |
| Interaction effects – a correlation between travel time and number of interactions in the main experiment | Gamma | ${log(\mathbb{E}[time}_{0j}])=\beta_{0}+\beta_{1}{interaction}_{0}+u_{j}$ |
| Interaction effects – a correlation between travel time and number of interactions in the control experiment 3 | Gamma | ${log(\mathbb{E}[time}_{3j}])=\beta_{0}+\beta_{1}{interaction}_{3}+u_{j}$ |
| Interaction effects – the number of interactions between the main experiment and control experiment 3 | Negative Binomial | ${log(\mathbb{E}[interaction}_{ij}])=\beta_{0}+\beta_{1}{experiment}_{i}+u_{j}$ |

**Table S1. GLMM information.**

**Table S2.** **The variables in the model equation and their descriptions in Figure S1.**

| **Variable** | **Description** |
| --- | --- |
| ${time}_{ij}$  ${angle}_{ij}$  ${speed}_{ij}$  ${interaction}_{ij}$ | Response variables for experiment/interaction *i* and colony 𝑗 |
| $\beta_{0}$ | Intercept |
| $\beta_{1}$ | Coefficient for the experiment/interaction variable |
| $u_{j}$ | Random effect for colony 𝑗 |


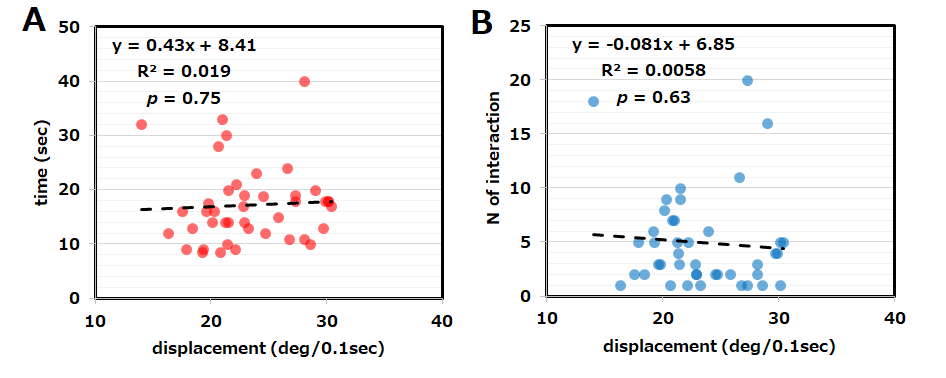


**Figure S3. Relationship between mean travel times (A) (or the number of interactions (B)) and angular displacement in the main experiment.** The dashed lines represent the regression lines.


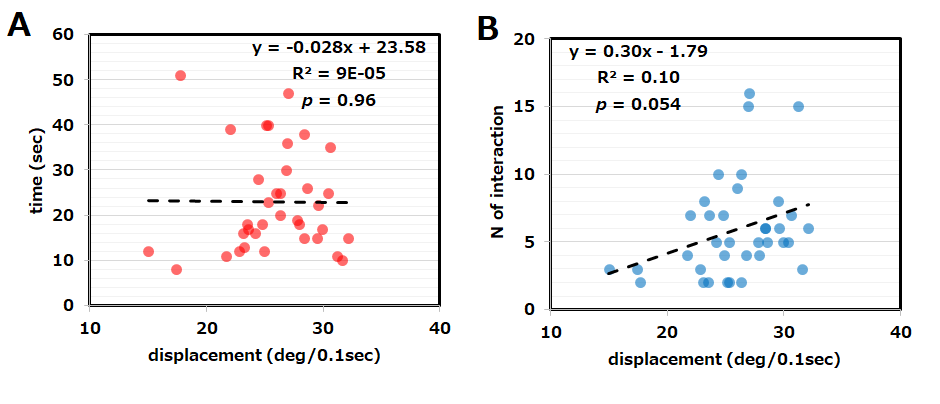


**Figure S4. A relationship between mean travel times (A) (or the number of interactions (B)) and angular displacement in the control experiment 3.** The dashed lines represent the regression lines.

**Analysis Script**

Time data analysis

> fit.glmer <- glmer(time ~ experiment + (1|colony), data = x, family = Gamma)

> summary(fit.glmer)

Generalized linear mixed model fit by maximum likelihood (Laplace Approximation) ['glmerMod']

Family: Gamma ( inverse )

Formula: time ~ experiment + (1 | colony)

Data: x

AIC BIC logLik deviance df.resid

1291.3 1310.2 -639.7 1279.3 165

Scaled residuals:

Min 1Q Median 3Q Max

-1.2367 -0.6773 -0.2580 0.4432 6.2557

Random effects:

Groups Name Variance Std.Dev.

colony (Intercept) 1.586e-05 0.003982

Residual 3.069e-01 0.553988

Number of obs: 171, groups: colony, 10

Fixed effects:

Estimate Std. Error t value Pr(>|z|)

(Intercept) 0.060447 0.005188 11.650 < 2e-16 ***

experimentB -0.016143 0.005299 -3.046 0.00232 **

experimentC -0.021817 0.005239 -4.164 3.12e-05 ***

experimentD -0.015668 0.005870 -2.669 0.00760 **

---

Signif. codes: 0 ‘***’ 0.001 ‘**’ 0.01 ‘*’ 0.05 ‘.’ 0.1 ‘ ’ 1

Correlation of Fixed Effects:

(Intr) exprmB exprmC

experimentB -0.707

experimentC -0.740 0.690

experimentD -0.741 0.626 0.635

> p_values <- c(0.00232, 3.12e-05, 0.00760)

> adjusted_p_values <- p.adjust(p_values, method = "BH")

> adjusted_p_values

[1] 3.48e-03 9.36e-05 7.60e-03

Angular displacement

> fit.glmer <- glmer(angle ~ experiment + (1|colony), data = x, family = Gamma)

boundary (singular) fit: see help('isSingular')

> summary(fit.glmer)

Generalized linear mixed model fit by maximum likelihood (Laplace

Approximation) [glmerMod]

Family: Gamma ( inverse )

Formula: angle ~ experiment + (1 | colony)

Data: x

AIC BIC logLik deviance df.resid

986.4 1005.3 -487.2 974.4 165

Scaled residuals:

Min 1Q Median 3Q Max

-2.3998 -0.6707 -0.1068 0.7668 2.7121

Random effects:

Groups Name Variance Std.Dev.

colony (Intercept) 0.00000 0.0000

Residual 0.03437 0.1854

Number of obs: 171, groups: colony, 10

Fixed effects:

Estimate Std. Error t value Pr(>|z|)

(Intercept) 0.042914 0.001245 34.474 < 2e-16 ***

experimentB 0.007096 0.001822 3.896 9.8e-05 ***

experimentC 0.003184 0.001816 1.753 0.0795 .

experimentD -0.004175 0.001739 -2.401 0.0163 *

---

Signif. codes: 0 ‘***’ 0.001 ‘**’ 0.01 ‘*’ 0.05 ‘.’ 0.1 ‘ ’ 1

Correlation of Fixed Effects:

(Intr) exprmB exprmC

experimentB -0.683

experimentC -0.685 0.468

experimentD -0.716 0.489 0.491

optimizer (Nelder_Mead) convergence code: 0 (OK)

boundary (singular) fit: see help('isSingular')

> isSingular(fit.glmer)

[1] TRUE

> fit.glm <- glm(angle ~ experiment, data = x, family = Gamma)

Angle displacement (part 2)

> summary(fit.glm)

Call:

glm(formula = angle ~ experiment, family = Gamma, data = x)

Coefficients:

Estimate Std. Error t value Pr(>|t|)

(Intercept) 0.042914 0.001242 34.547 < 2e-16 ***

experimentB 0.007096 0.001817 3.904 0.000137 ***

experimentC 0.003184 0.001812 1.757 0.080694 .

experimentD -0.004175 0.001735 -2.407 0.017195 *

---

Signif. codes: 0 ‘***’ 0.001 ‘**’ 0.01 ‘*’ 0.05 ‘.’ 0.1 ‘ ’ 1

(Dispersion parameter for Gamma family taken to be 0.03519098)

Null deviance: 7.5724 on 170 degrees of freedom

Residual deviance: 6.0828 on 167 degrees of freedom

AIC: 982.44

Number of Fisher Scoring iterations: 4

> p_values <- c(0.000137, 0.080694, 0.017195)

> adjusted_p_values <- p.adjust(p_values, method = "BH")

> adjusted_p_values

[1] 0.0004110 0.0806940 0.0257925

Speed

> fit.glmer <- glmer(speed ~ experiment + (1|colony), data = x, family = Gamma)

> summary(fit.glmer)

Generalized linear mixed model fit by maximum likelihood (Laplace

Approximation) [glmerMod]

Family: Gamma ( inverse )

Formula: speed ~ experiment + (1 | colony)

Data: x

AIC BIC logLik deviance df.resid

-405.5 -386.6 208.7 -417.5 165

Scaled residuals:

Min 1Q Median 3Q Max

-2.2302 -0.6816 -0.0669 0.5299 3.5605

Random effects:

Groups Name Variance Std.Dev.

colony (Intercept) 0.06237 0.2497

Residual 0.12713 0.3566

Number of obs: 171, groups: colony, 10

Fixed effects:

Estimate Std. Error t value Pr(>|z|)

(Intercept) 4.1393 0.3111 13.307 < 2e-16 ***

experimentB 0.9577 0.3632 2.637 0.008371 **

experimentC 1.3571 0.3956 3.430 0.000603 ***

experimentD 0.6978 0.4176 1.671 0.094771 .

---

Signif. codes: 0 ‘***’ 0.001 ‘**’ 0.01 ‘*’ 0.05 ‘.’ 0.1 ‘ ’ 1

Correlation of Fixed Effects:

(Intr) exprmB exprmC

experimentB -0.537

experimentC -0.488 0.389

experimentD -0.626 0.405 0.340

> p_values <- c(0.008371, 0.000603, 0.094771)

> adjusted_p_values <- p.adjust(p_values, method = "BH")

> adjusted_p_values

[1] 0.0125565 0.0018090 0.0947710

Time ~ Interaction - Main

> fit.glmer <- glmer(time ~ interaction + (1|colony), data = x, family = Gamma)

Warning message:

1: checkConv(attr(opt, "derivs"), opt$par, ctrl = control$checkConv,

Model failed to converge with max|grad| = 0.00635482 (tol = 0.002, component 1)

2: checkConv(attr(opt, "derivs"), opt$par, ctrl = control$checkConv,

Model is nearly unidentifiable: very large eigenvalue

- Rescale variables?

> summary(fit.glmer)

Generalized linear mixed model fit by maximum likelihood (Laplace

Approximation) [glmerMod]

Family: Gamma ( inverse )

Formula: time ~ interaction + (1 | colony)

Data: x

AIC BIC logLik deviance df.resid

277.5 284.4 -134.7 269.5 38

Scaled residuals:

Min 1Q Median 3Q Max

-1.3057 -0.5844 -0.1784 0.3824 3.4153

Random effects:

Groups Name Variance Std.Dev.

colony (Intercept) 3.651e-05 0.006042

Residual 1.501e-01 0.387435

Number of obs: 42, groups: colony, 4

Fixed effects:

Estimate Std. Error t value Pr(>|z|)

(Intercept) 0.0668967 0.0068014 9.836 <2e-16 ***

interaction -0.0010583 0.0005894 -1.796 0.0726 .

---

Signif. codes: 0 ‘***’ 0.001 ‘**’ 0.01 ‘*’ 0.05 ‘.’ 0.1 ‘ ’ 1

Correlation of Fixed Effects:

(Intr)

interaction -0.465

optimizer (Nelder_Mead) convergence code: 0 (OK)

Model failed to converge with max|grad| = 0.013902 (tol = 0.002, component 1)

Model is nearly unidentifiable: very large eigenvalue

- Rescale variables?

> isSingular(fit.glmer)

[1] FALSE

> x$interaction <- scale(x$interaction)

> fit.glmer <- glmer(time ~ interaction + (1|colony), data = x, family = Gamma)

Time ~ Interaction – Main (part 2)

> summary(fit.glmer)

Generalized linear mixed model fit by maximum likelihood (Laplace

Approximation) [glmerMod]

Family: Gamma ( inverse )

Formula: time ~ interaction + (1 | colony)

Data: x

AIC BIC logLik deviance df.resid

277.5 284.4 -134.7 269.5 38

Scaled residuals:

Min 1Q Median 3Q Max

-1.3057 -0.5844 -0.1784 0.3824 3.4152

Random effects:

Groups Name Variance Std.Dev.

colony (Intercept) 3.651e-05 0.006043

Residual 1.501e-01 0.387433

Number of obs: 42, groups: colony, 4

Fixed effects:

Estimate Std. Error t value Pr(>|z|)

(Intercept) 0.061656 0.006026 10.232 <2e-16 ***

interaction -0.004818 0.002690 -1.791 0.0733 .

---

Signif. codes: 0 ‘***’ 0.001 ‘**’ 0.01 ‘*’ 0.05 ‘.’ 0.1 ‘ ’ 1

Correlation of Fixed Effects:

(Intr)

interaction -0.043

Time ~ Interaction – Control-3

> fit.glmer <- glmer(time ~ interaction + (1|colony), data = x, family = Gamma)

Warning message:

1: checkConv(attr(opt, "derivs"), opt$par, ctrl = control$checkConv,

Model failed to converge with max|grad| = 0.00403591 (tol = 0.002, component 1)

2: checkConv(attr(opt, "derivs"), opt$par, ctrl = control$checkConv,

Model is nearly unidentifiable: very large eigenvalue

- Rescale variables?

> summary(fit.glmer)

Generalized linear mixed model fit by maximum likelihood (Laplace

Approximation) [glmerMod]

Family: Gamma ( inverse )

Formula: time ~ interaction + (1 | colony)

Data: x

AIC BIC logLik deviance df.resid

274.4 280.8 -133.2 266.4 32

Scaled residuals:

Min 1Q Median 3Q Max

-1.2609 -0.6545 -0.2363 0.4680 2.8257

Random effects:

Groups Name Variance Std.Dev.

colony (Intercept) 4.714e-05 0.006866

Residual 2.163e-01 0.465051

Number of obs: 36, groups: colony, 10

Fixed effects:

Estimate Std. Error t value Pr(>|z|)

(Intercept) 0.0511147 0.0069209 7.386 1.52e-13 ***

interaction -0.0009485 0.0007765 -1.221 0.222

---

Signif. codes: 0 ‘***’ 0.001 ‘**’ 0.01 ‘*’ 0.05 ‘.’ 0.1 ‘ ’ 1

Correlation of Fixed Effects:

(Intr)

interaction -0.677

optimizer (Nelder_Mead) convergence code: 0 (OK)

Model failed to converge with max|grad| = 0.00403591 (tol = 0.002, component 1)

Model is nearly unidentifiable: very large eigenvalue

- Rescale variables?

> isSingular(fit.glmer)

[1] FALSE

> x$interaction <- scale(x$interaction)

> fit.glmer <- glmer(time ~ interaction + (1|colony), data = x, family = Gamma)

Time ~ Interaction – Control-3 (part 2)

> summary(fit.glmer)

Generalized linear mixed model fit by maximum likelihood (Laplace

Approximation) [glmerMod]

Family: Gamma ( inverse )

Formula: time ~ interaction + (1 | colony)

Data: x

AIC BIC logLik deviance df.resid

274.4 280.8 -133.2 266.4 32

Scaled residuals:

Min 1Q Median 3Q Max

-1.2609 -0.6545 -0.2363 0.4681 2.8257

Random effects:

Groups Name Variance Std.Dev.

colony (Intercept) 4.714e-05 0.006866

Residual 2.163e-01 0.465052

Number of obs: 36, groups: colony, 10

Fixed effects:

Estimate Std. Error t value Pr(>|z|)

(Intercept) 0.045503 0.005096 8.929 <2e-16 ***

interaction -0.003471 0.002861 -1.213 0.225

---

Signif. codes: 0 ‘***’ 0.001 ‘**’ 0.01 ‘*’ 0.05 ‘.’ 0.1 ‘ ’ 1

Correlation of Fixed Effects:

(Intr)

interaction -0.022

Interaction – Main vs. Control-3

> fit.nb <- glmer(interaction ~ experiment + (1|colony), data = x, family = negative.binomial(1))

boundary (singular) fit: see help('isSingular')

> summary(fit.nb)

Generalized linear mixed model fit by maximum likelihood (Laplace

Approximation) [glmerMod]

Family: Negative Binomial(1) ( log )

Formula: interaction ~ experiment + (1 | colony)

Data: x

AIC BIC logLik deviance df.resid

440.1 449.5 -216.1 432.1 74

Scaled residuals:

Min 1Q Median 3Q Max

-0.7280 -0.5438 -0.1433 0.1870 2.7715

Random effects:

Groups Name Variance Std.Dev.

colony (Intercept) 1.073e-17 3.276e-09

Number of obs: 78, groups: colony, 10

Fixed effects:

Estimate Std. Error z value Pr(>|z|)

(Intercept) 1.5999 0.1692 9.457 <2e-16 ***

experimentD 0.1779 0.2472 0.720 0.472

---

Signif. codes: 0 ‘***’ 0.001 ‘**’ 0.01 ‘*’ 0.05 ‘.’ 0.1 ‘ ’ 1

Correlation of Fixed Effects:

(Intr)

experimentD -0.684

optimizer (Nelder_Mead) convergence code: 0 (OK)

boundary (singular) fit: see help('isSingular')

> isSingular(fit.nb)

[1] TRUE

> fit.nb.fixed <- glm(interaction ~ experiment, data = x, family = negative.binomial(1))

> summary(fit.nb.fixed)

Call:

glm(formula = interaction ~ experiment, family = negative.binomial(1),

data = x)

Deviance Residuals:

Min 1Q Median 3Q Max

-1.0783 -0.6958 -0.1508 0.1764 1.6982

Coefficients:

Estimate Std. Error t value Pr(>|t|)

(Intercept) 1.5999 0.1232 12.990 <2e-16 ***

experimentD 0.1779 0.1800 0.989 0.326

---

Signif. codes: 0 ‘***’ 0.001 ‘**’ 0.01 ‘*’ 0.05 ‘.’ 0.1 ‘ ’ 1

(Dispersion parameter for Negative Binomial(1) family taken to be 0.5300744)

Null deviance: 34.589 on 77 degrees of freedom

Residual deviance: 34.070 on 76 degrees of freedom

AIC: 436.12

Number of Fisher Scoring iterations: 5

Time data analysis including ants’ data that did not reach the feeder

> fit.glmer <- glmer(time ~ experiment + (1|colony), data = x, family = Gamma)

> summary(fit.glmer)

Generalized linear mixed model fit by maximum likelihood (Laplace

Approximation) [glmerMod]

Family: Gamma ( inverse )

Formula: time ~ experiment + (1 | colony)

Data: x

AIC BIC logLik deviance df.resid

1336.1 1355.2 -662.1 1324.1 171

Scaled residuals:

Min 1Q Median 3Q Max

-1.2495 -0.6878 -0.2676 0.4584 6.2450

Random effects:

Groups Name Variance Std.Dev.

colony (Intercept) 1.616e-05 0.00402

Residual 3.042e-01 0.55152

Number of obs: 177, groups: colony, 10

Fixed effects:

Estimate Std. Error t value Pr(>|z|)

(Intercept) 0.059850 0.004984 12.007 < 2e-16 ***

experimentB -0.015560 0.005148 -3.023 0.00251 **

experimentC -0.021230 0.005080 -4.179 2.92e-05 ***

experimentD -0.015466 0.005697 -2.715 0.00664 **

---

Signif. codes: 0 ‘***’ 0.001 ‘**’ 0.01 ‘*’ 0.05 ‘.’ 0.1 ‘ ’ 1

Correlation of Fixed Effects:

(Intr) exprmB exprmC

experimentB -0.687

experimentC -0.720 0.668

experimentD -0.736 0.604 0.615

> p_values <- c(0.00251, 2.92e-05, 0.00664)

> adjusted_p_values <- p.adjust(p_values, method = "BH")

> adjusted_p_values

[1] 0.0037650 0.0000876 0.0066400
